# Remarks *on Reading Mr. Ward's Theory of the Modus Operandi of Opium; Communicated to Doctor Bradley*

**Published:** 1803-02-01

**Authors:** George Nesse Hill

**Affiliations:** Chester


### Remarks *on reading Mr. Ward's Theory of the Modus Operandi of Opium; communicated to Doctor Bradley by* Mr. George Nesse Hill, *of Chester.*

Having taken some pains to understand the New Theory of the Modus Operandi of Opium, as attempted to be established by Mr. Ward of Manchester, and finding that every number of your useful work, containing any of that gentleman's remarks on this great subject, has tended only to convince me of the just foundation in truth of Dr. Crumpe's statement, (although that author's work has not fallen into my hands) I have taken the liberty of troubling you with a few occasional thoughts, which have occurred to me on this important matter; if you think the best interests of our profession will in any degree be furthered by by the insertion of them, I crave a place in the Journal, at your convenience; if not, please to set them aside.

I have, for some time, been waiting with some degree of impatience, to see the doctrine Mr. W. has endeavoured to elucidate, and enforce, freely canvassed and discussed in all its views, that its harmony with truth, and its consistency with experience, might establish the fact, or its fallacy and speciousness be detected, and the question be for ever set at rest. Hitherto I have been disappointed; it will give me no trifling satisfaction to find that what I have said has tended to the promotion of this desirable end of all amicable discussion, being long since assured that the real interests of truth have nothing to apprehend from the closest severity of investigation, nor the utmost censure human judgment can pronounce. In common with the rest of your readers, I feel grateful for the ready and accurate communication Mr. W. has given the Medical World in his several instructive papers on the external uses of opium; permit me to add my feeble testimony to the excellence of the practice; nor have I a doubt but his name will be remembered with sincere gratitude by many an unhappy sufferer, who is condemned to the daily use of this medicine. It does not appear to me that this gentleman, when he first announced this matter to the public, conceived of the action of opium in the manner he has lately avowed, when employed in his critique on Dr. Crumpe's Inquiry; for in some of his first communications he speaks of this drug as a tonic stimulant, in the later ones as a sedative. Now one or other of these terms, by whomsoever adopted and acted upon, will certainly lead to false conclusions, and ought to be abandoned, unless he can shew that the medicine possesses opposite qualities, or produces opposite effects from the manner of using it. Perhaps Mr. W. could scarce have selected from important medical authors, a stronger proof of the great tonic stimulant powers of opium than that given by the celebrated Mr. Pott in his Treatise on the Mortification of the Toes and Feet; a quotation from which Mr. W. has chosen for the commencement of his observations. It would be an improper disposal of time, to bring any proofs to shew that the disease, which Mr. Pott so admirably treated, was one of those arising from and dependant upon debility; the history he has given of the subjects of its attack, the symptoms of the disease, and its usual termination, it is sufficient barely to mention. The effects of well known and acknowledged knowledged stimulants, as the bark, snake root, aromatics, volatile salts, and musk, in retarding the disease, are so many witnesses that a higher or more powerful tonic stimulant was wanting. For the purpose of easing pain, opium was administered. Does any one doubt the cause of this symptom being an hourly increasing destructive debility? And will any one hesitate in denying that a powerful sedative must not have increased the pain, by destroying vigor, or debilitating still more? No one, I believe, would chuse to try the experiment of exhibiting evacuants and debilitants on such an occasion; consequently, no one, thinking thus, would refuse to acknowledge opium as possessing very opposite qualities. But it is not improbable that Mr. Ward's ideas of the definition of the words stimulant and sedative, are very different to mine; if it should hereafter appear they are almost in direct opposition to each other, this circumstance will account for every thing. This point once settled, the next question is, to which of these classes of powerful remedies does opium belong? We are not favoured with Mr. W's definitions of these important words, until we arrive at page 127 of Number xxxvi; and here I may be permitted to observe, that the accusation brought against the advocates for the stimulant doctrine, as Mr. W. calls it, is not less ascribable to those of an opposite opinion, this gentleman sometimes calling opium a tonic, at others a sedative; hence from nothing he, or those authors he has quoted, have said, is the precise definition of the two terms better settled, at least as far as regards opium, although the next page gives us Mr. W's precise meaning, adopted from Dr. Cullen's system; but in this very important instance, the fallibility of this system is, in my humble opinion, glaringly evident. Dr. C. says, " Stimulants are powers capable of increasing the mobility, and of exciting the motion of the nervous power." He must here mean that the nerves, their power, mobility, &c. are the organs by which every thing in the body is influenced, and upon which all muscular action is dependant; and that whatever has the faculty of increasing the force, or energy, of this action or power, will produce a proportionate degree of tone and vigor of the part acted upon primarily, and secondarily of the whole system; but do we not every day perceive that great readiness to action may be produced without great power to support it? Every stimulating agent therefore cannot be denominated a tonic; hence we have numerous substances which will stimulate, and produce increased sensibility and irritability, as they are called, without giving vigor or imparting tone to the body; but it has not yet appeared to me that opium belongs to this class, for its modus operandi, in my opinion, is to impart vigor, and remove morbid sensibility and irritability, inasmuch as these states are dependant upon debility; and this I understand as a just definition of all tonic stimulants. Sedatives he defines as "medicines which diminish the sensibility and irritability of the system, and thereby the motion and powers of motion in it." I humbly presume a little transposition of terms might here be reasonably allowed, without taking too great a liberty with the text of the learned Nosologist; I will venture to say, then, that as sensibility and irritability are states dependent on the exact force of action existing in the body at the time of their being too great or too little, it is an increase or diminution of this state which must increase or diminish those that are dependant thereon; the term sedative I can never understand as conveying any other idea, than that of a power calculated by certain inherent properties to lessen the tone or vigor of the animal system, and all the symptoms consequent on too energetic action; therefore when one author in his definition, says, they are powers which diminish sensibility and irritability, are adapted to allay inordinate motion of the muscular fibres, or to tranquillize and calm the irritated nerves, &c. we can only lament that the writer should have been so vague and indefinite. I infer, that whilst this remains the case on subjects of such incalculable importance, it is almost in vain for writers to argue, or endeavour to explain away the meanings of each other, seeing they have no good and solid ground to rest upon; all the various powerful medicines that are hourly applied to the human frame, must fall under one or other of the classes now mentioned; seeing that whatever is applied to it, whether externally or internally, must, either sufficiently or insufficiently, mechanically or chemically, stimulate and increase, or diminish and destroy its power of action. Hence was it well said by the late Dr. Maddocks, that, "in framing a definition, we should select such circumstances appertaining to the matter to be defined, as are essential, absolutely necessary, always uniform and clear, admitting no abstruse reasoning, incapable of more than one meaning, (not containing two meanings conveying the most opposite construction) and, finally, as easy to be comprehended by the illiterate as the learned." How would Mr. W. attempt to make make a person, unacquainted with physical terms, comprehend the meaning of the words Stimulant and Sedative? It appears to me he would explain them in some such way as the following: "If you take a large dose of Glauber's salts, and it freely purges you, the effect produced is a temporary weakness brought on you by the Sedative power of the salts. If you are about to make some great exertion, as lifting a heavy load, to which you are scarce equal, but by drinking a glass of brandy are enabled to raise it; you have derived this additional, though momentary strength, from a stimulant. I apprehend, that upon further consideration, Mr. W. must concede to Dr. Brown that neutral salts are, notwithstanding, stimulants, producing their effects by increasing the action of the stomach and bowels; but although they do this, shall I be told they also increase the tone of these parts? I presume not; the fair inference then is, that whatever increases the power of acting, is alone justly entitled to the name of a tonic, and that the term stimulant should be left out of the consideration, because although all substances foreign to the body do stimulate when applied to its different organs, yet great numbers of them diminish power; and, strictly speaking, whatever does so, can alone be termed a sedative. But, as already observed, Mr. W. has applied both these terms to opium; it is evident one or other of them must be abandoned, unless he can by any means prove that the medicine possesses opposite qualities, or has diametrically opposite effects, according to the mode of its exhibition.

*December* 31, 1802.

